# Supplementary material for: Barriers and facilitators to the conduct of critical care research in low and lower-middle income countries: A scoping review
Source: PLoS One. 2022 May 5;17(5):e0266836. doi: 10.1371/journal.pone.0266836 (PMC9071139; doi:10.1371/journal.pone.0266836)
Supplement: S1 Appendix — (DOCX) [file pone.0266836.s001.docx]

**Online supplementary material: Search strategy:^*^**

Database: Ovid MEDLINE(R) and Epub Ahead of Print, In-Process & Other Non-Indexed Citations, Daily and Versions(R) <1946 to August 26, 2020> Search Strategy:

--------------------------------------------------------------------------------

1 (barrier* adj10 (research* or study or studies or trial*)).ti,ab,kf,hw. (34402)

2 (challenge* adj10 (research* or study or studies or trial*)).ti,ab,kf,hw. (74568)

3 (obstacle* adj10 (research* or study or studies or trial*)).ti,ab,kf,hw. (5143)

4 or/1-3 (112218)

5 exp Critical Care/ (58129)

6 critical care.mp. (72546)

7 exp Critical Illness/ (29229)

8 critical* ill*.mp. (60664)

9 exp Intensive Care Units/ (85074)

10 (intensive care or ICU*).mp. (192459)

11 exp Respiratory Distress Syndrome, Adult/ (19651)

12 acute respiratory distress syndrome*.mp. (14463)

13 exp Respiratory Insufficiency/ (62682)

14 respiratory failure*.mp. (31773)

15 exp Acute Kidney Injury/ (46495)

16 acute kidney injur*.mp. (55781)

17 exp Multiple Organ Failure/ (10921)

18 multi* organ* failure*.mp. (18248)

19 exp Liver Failure/ (25372)

20 liver failure*.mp. (25630)

21 exp Renal Insufficiency/ (173261)

22 ((renal or kidney*) adj1 failure*).mp. (164646)

23 exp Sepsis/ (123609)

24 sepsis.mp. (126208)

25 exp Shock/ (75101)

26 shock*.mp. (238895)

27 exp "Wounds and Injuries"/ (905660)

28 trauma.mp. (267889)

29 exp Snake Bites/ (4644)

30 (snakebite* or snake bite*).mp. (6109)

31 exp Poisoning/ (157691)

32 poison*.mp. (158975)

33 exp Tropical Medicine/ (5901)

34 tropical disease*.mp. (6125)

35 or/5-34 [critical care or common conditions in critical care] (2120923)

36 exp Research/ (623381)

37 research.mp. (10447829)

38 exp Clinical Study/ (956352)

39 exp Clinical Trial/ (867744)

40 exp Clinical Trials as Topic/ (344948)

41 exp Research Support as Topic/ (22548)

42 (trial* or study or studies).mp. (12329524)

43 or/36-42 [research or clinical studies] (17161443)

44 Angola.cp,ti,ab,in,hw. (1841)

45 Bangladesh.cp,ti,ab,in,hw. (24946)

46 Bhutan.cp,ti,ab,in,hw. (938)

47 Bolivia.cp,ti,ab,in,hw. (5168)

48 Cabo Verde.cp,ti,ab,in,hw. (302)

49 Cambodia.cp,ti,ab,in,hw. (5551)

50 Cameroon.cp,ti,ab,in,hw. (11228)

51 Comoros.cp,ti,ab,in,hw. (519)

52 Congo.cp,ti,ab,in,hw. (17148)

53 Djibouti.cp,ti,ab,in,hw. (487)

54 Egypt.cp,ti,ab,in,hw. (123317)

55 El Salvador.cp,ti,ab,in,hw. (2139)

56 Eswatini.cp,ti,ab,in,hw. (684)

57 Ghana.cp,ti,ab,in,hw. (16105)

58 Honduras.cp,ti,ab,in,hw. (2610)

59 India.cp,ti,ab,in,hw. (748158)

60 Indonesia.cp,ti,ab,in,hw. (27137)

61 Kenya.cp,ti,ab,in,hw. (38367)

62 Kiribati.cp,ti,ab,in,hw. (208)

63 (Kyrgyzstan or Kyrgyz Republic).cp,ti,ab,in,hw. (2677)

64 (Laos or Lao peoples democratic republic).cp,ti,ab,in,hw. (3264)

65 Lesotho.cp,ti,ab,in,hw. (844)

66 Mauritania.cp,ti,ab,in,hw. (784)

67 Micronesia.cp,ti,ab,in,hw. (1555)

68 Moldova.cp,ti,ab,in,hw. (1720)

69 Mongolia.cp,ti,ab,in,hw. (11055)

70 Morocco.cp,ti,ab,in,hw. (19225)

71 Myanmar.cp,ti,ab,in,hw. (4831)

72 Nicaragua.cp,ti,ab,in,hw. (2601)

73 Nigeria.cp,ti,ab,in,hw. (58558)

74 Pakistan.cp,ti,ab,in,hw. (71808)

75 (Papua New Guinea or East New Guinea).cp,ti,ab,in,hw. (6159)

76 Philippines.cp,ti,ab,in,hw. (20669)

77 Senegal.cp,ti,ab,in,hw. (12303)

78 (Solomon Islands or Melanesia).cp,ti,ab,in,hw. (1661)

79 Sudan.cp,ti,ab,in,hw. (12187)

80 Timor-Leste.cp,ti,ab,in,hw. (455)

81 Tunisia.cp,ti,ab,in,hw. (29430)

82 Ukraine.cp,ti,ab,in,hw. (98351)

83 Uzbekistan.cp,ti,ab,in,hw. (3432)

84 Vanuatu.cp,ti,ab,in,hw. (759)

85 Vietnam.cp,ti,ab,in,hw. (26072)

86 Zambia.cp,ti,ab,in,hw. (7660)

87 Zimbabwe.cp,ti,ab,in,hw. (12391)

88 or/44-87 [World Bank Lower-Middle Income Economies] (1364059)

89 Afghanistan.cp,ti,ab,in,hw. (7195)

90 Benin.cp,ti,ab,in,hw. (6232)

91 Burkina Faso.cp,ti,ab,in,hw. (5968)

92 Burundi.cp,ti,ab,in,hw. (1118)

93 Central African Republic.cp,ti,ab,in,hw. (1303)

94 Chad.cp,ti,ab,in,hw. (10301)

95 Democratic Republic of the Congo.cp,ti,ab,in,hw. (5037)

96 Eritrea.cp,ti,ab,in,hw. (1079)

97 Ethiopia.cp,ti,ab,in,hw. (22548)

98 Gambia.cp,ti,ab,in,hw. (4163)

99 Guniea.cp,ti,ab,in,hw. (113)

100 Guinea-Bissau.cp,ti,ab,in,hw. (1409)

101 Haiti.cp,ti,ab,in,hw. (4402)

102 (Democratic People's Republic of Korea or North Korea).cp,ti,ab,in,hw. (635)

103 Liberia.cp,ti,ab,in,hw. (2091)

104 Madagascar.cp,ti,ab,in,hw. (5915)

105 Malawi.cp,ti,ab,in,hw. (9669)

106 Mali.cp,ti,ab,in,hw. (6220)

107 Mozambique.cp,ti,ab,in,hw. (4773)

108 Nepal.cp,ti,ab,in,hw. (17179)

109 Niger.cp,ti,ab,in,hw. (14480)

110 Rwanda.cp,ti,ab,in,hw. (4289)

111 Sierra Leone.cp,ti,ab,in,hw. (2805)

112 Somalia.cp,ti,ab,in,hw. (2314)

113 South Sudan.cp,ti,ab,in,hw. (627)

114 (Syrian Arab Republic or Syria).cp,ti,ab,in,hw. (4537)

115 Tajikistan.cp,ti,ab,in,hw. (1139)

116 Tanzania.cp,ti,ab,in,hw. (18539)

117 Togo.cp,ti,ab,in,hw. (2724)

118 Uganda.cp,ti,ab,in,hw. (28328)

119 Yemen.cp,ti,ab,in,hw. (3192)

120 or/89-119 [World Bank Low income economies] (182548)

121 88 or 120 [World Bank Low or Lower Middle Income Economies 2019 2020] (1500863)

122 4 and 35 and 43 and 121 (476)

123 limit 122 to english language (469)

***************************

Database: Embase Classic+Embase <1947 to 2020 Week 34> Search Strategy:

--------------------------------------------------------------------------------

1 (barrier* adj10 (research* or study or studies or trial*)).ti,ab,kw,hw. (49974)

2 (challenge* adj10 (research* or study or studies or trial*)).ti,ab,kw,hw. (93807)

3 (obstacle* adj10 (research* or study or studies or trial*)).ti,ab,kw,hw. (6558)

4 or/1-3 (147858)

5 critical care.ti,ab,kw,hw. (54901)

6 exp critical illness/ (29725)

7 critical* ill*.ti,ab,kw,hw. (96836)

8 exp intensive care unit/ (198155)

9 (intensive care or ICU*).ti,ab,kw,hw. (398693)

10 exp adult respiratory distress syndrome/ (38124)

11 acute respiratory distress syndrome*.ti,ab,kw,hw. (21383)

12 exp respiratory failure/ (108736)

13 respiratory failure*.ti,ab,kw,hw. (103796)

14 exp acute kidney failure/ (90349)

15 acute kidney injur*.ti,ab,kw,hw. (39461)

16 exp multiple organ failure/ (40480)

17 multi* organ* failure*.ti,ab,kw,hw. (45378)

18 exp liver failure/ (79231)

19 liver failure*.ti,ab,kw,hw. (62473)

20 exp kidney failure/ (392173)

21 ((renal or kidney) adj1 failure*).ti,ab,kw,hw. (355521)

22 exp sepsis/ (280342)

23 sepsis.ti,ab,kw,hw. (224142)

24 exp shock/ (147759)

25 shock*.ti,ab,kw,hw. (372235)

26 exp injury/ (2416977)

27 trauma.ti,ab,kw,hw. (360823)

28 exp snakebite/ (6852)

29 (snake bite* or snakebite*).ti,ab,kw,hw. (8058)

30 exp intoxication/ (428750)

31 poisoning.ti,ab,kw,hw. (120507)

32 exp tropical medicine/ (17643)

33 exp tropical disease/ (183410)

34 tropical disease.ti,ab,kw,hw. (6877)

35 or/5-34 (4354966)

36 exp research/ (827382)

37 research.ti,ab,kw,hw. (2647347)

38 exp clinical research/ (81403)

39 exp medical research/ (425729)

40 exp clinical study/ (10127710)

41 exp clinical trial/ (1536104)

42 exp "clinical trial (topic)"/ (330118)

43 (trial* or study or studies).ti,ab,kw,hw. (19568891)

44 or/36-43 (23141421)

45 Angola.cp,ti,ab,in,hw. (2347)

46 Bangladesh.cp,ti,ab,in,hw. (36527)

47 Bhutan.cp,ti,ab,in,hw. (1169)

48 Bolivia.cp,ti,ab,in,hw. (6498)

49 Cabo Verde.cp,ti,ab,in,hw. (152)

50 Cambodia.cp,ti,ab,in,hw. (7331)

51 Cameroon.cp,ti,ab,in,hw. (15193)

52 Comoros.cp,ti,ab,in,hw. (554)

53 Congo.cp,ti,ab,in,hw. (24156)

54 Djibouti.cp,ti,ab,in,hw. (655)

55 Egypt.cp,ti,ab,in,hw. (147477)

56 El Salvador.cp,ti,ab,in,hw. (3533)

57 Eswatini.cp,ti,ab,in,hw. (144)

58 Ghana.cp,ti,ab,in,hw. (20936)

59 Honduras.cp,ti,ab,in,hw. (3375)

60 India.cp,ti,ab,in,hw. (1055576)

61 Indonesia.cp,ti,ab,in,hw. (51363)

62 Kenya.cp,ti,ab,in,hw. (50769)

63 Kiribati.cp,ti,ab,in,hw. (234)

64 (Kyrgyzstan or Kyrgyz Republic).cp,ti,ab,in,hw. (2052)

65 (Laos or Lao peoples democratic republic).cp,ti,ab,in,hw. (4089)

66 Lesotho.cp,ti,ab,in,hw. (1116)

67 Mauritania.cp,ti,ab,in,hw. (1038)

68 Micronesia.cp,ti,ab,in,hw. (1447)

69 Moldova.cp,ti,ab,in,hw. (3398)

70 Mongolia.cp,ti,ab,in,hw. (15039)

71 Morocco.cp,ti,ab,in,hw. (32302)

72 Myanmar.cp,ti,ab,in,hw. (6067)

73 Nicaragua.cp,ti,ab,in,hw. (3414)

74 Nigeria.cp,ti,ab,in,hw. (93987)

75 Pakistan.cp,ti,ab,in,hw. (113751)

76 (Papua New Guinea or East New Guinea).cp,ti,ab,in,hw. (9271)

77 Philippines.cp,ti,ab,in,hw. (30047)

78 Senegal.cp,ti,ab,in,hw. (16484)

79 (Solomon Islands or Melanesia).cp,ti,ab,in,hw. (1994)

80 Sudan.cp,ti,ab,in,hw. (17242)

81 Timor-Leste.cp,ti,ab,in,hw. (753)

82 Tunisia.cp,ti,ab,in,hw. (49478)

83 Ukraine.cp,ti,ab,in,hw. (76587)

84 Uzbekistan.cp,ti,ab,in,hw. (5906)

85 Vanuatu.cp,ti,ab,in,hw. (829)

86 Vietnam.cp,ti,ab,in,hw. (31000)

87 Zambia.cp,ti,ab,in,hw. (10200)

88 Zimbabwe.cp,ti,ab,in,hw. (13880)

89 or/45-88 [World Bank Lower-Middle Income Economies] (1840692)

90 Afghanistan.cp,ti,ab,in,hw. (9358)

91 Benin.cp,ti,ab,in,hw. (10190)

92 Burkina Faso.cp,ti,ab,in,hw. (8129)

93 Burundi.cp,ti,ab,in,hw. (1334)

94 Central African Republic.cp,ti,ab,in,hw. (1416)

95 Chad.cp,ti,ab,in,hw. (2447)

96 Democratic Republic of the Congo.cp,ti,ab,in,hw. (1458)

97 Eritrea.cp,ti,ab,in,hw. (3569)

98 Ethiopia.cp,ti,ab,in,hw. (25692)

99 Gambia.cp,ti,ab,in,hw. (5444)

100 Guniea.cp,ti,ab,in,hw. (45)

101 Guinea-Bissau.cp,ti,ab,in,hw. (1645)

102 Haiti.cp,ti,ab,in,hw. (5400)

103 (Democratic People's Republic of Korea or North Korea).cp,ti,ab,in,hw. (2727)

104 Liberia.cp,ti,ab,in,hw. (2791)

105 Madagascar.cp,ti,ab,in,hw. (7243)

106 Malawi.cp,ti,ab,in,hw. (11947)

107 Mali.cp,ti,ab,in,hw. (8880)

108 Mozambique.cp,ti,ab,in,hw. (6216)

109 Nepal.cp,ti,ab,in,hw. (21889)

110 Niger.cp,ti,ab,in,hw. (23662)

111 Rwanda.cp,ti,ab,in,hw. (5586)

112 Sierra Leone.cp,ti,ab,in,hw. (3499)

113 Somalia.cp,ti,ab,in,hw. (2683)

114 South Sudan.cp,ti,ab,in,hw. (741)

115 (Syrian Arab Republic or Syria).cp,ti,ab,in,hw. (6471)

116 Tajikistan.cp,ti,ab,in,hw. (1404)

117 Tanzania.cp,ti,ab,in,hw. (23488)

118 Togo.cp,ti,ab,in,hw. (4494)

119 Uganda.cp,ti,ab,in,hw. (31341)

120 Yemen.cp,ti,ab,in,hw. (4172)

121 or/90-120 [World Bank Low income economies] (221196)

122 89 or 121 [World Bank Low or Lower Middle Income Economies 2019 2020] (2001154)

123 4 and 35 and 44 and 122 (1635)

124 limit 123 to english language (1624)

***************************

^*Same search strategy was used when we extended the search to December 2021.^
