# Supplementary material for: Barriers and facilitators to the conduct of critical care research in low and lower-middle income countries: A scoping review
Source: PLoS One. 2022 May 5;17(5):e0266836. doi: 10.1371/journal.pone.0266836 (PMC9071139; doi:10.1371/journal.pone.0266836)
Supplement: S2 Appendix — (DOCX) [file pone.0266836.s002.docx]

**Online Supplementary material- Study quality assessment: For the below studies, as they all used a qualitative approach, the tool proposed by Kuper et al^17^  has been used for quality assessment.**

1. **Study quality assessment: Ahmed et al.**

| **Appraisal questions** | **Yes** | **No** | **Unsure** | **NA** | **Comments** |
| --- | --- | --- | --- | --- | --- |
|  |  |  |  |  |  |
| Was the sample used appropriate for the research question?  Consider if the sample was chosen keeping in mind participant experiences as relevant to the study question, but also based on social affiliations- for e.g. religion, culture, profession etc. |  |  |  |  | Participants were researchers from LMICs attending a workshop (group discussion) focused on barriers to research/researchers returning home.  While the population chosen is appropriate, a detailed description of participants is lacking. |
| Were the data collected appropriately?  Consider the following:   - Were the methods for data collection described? - Were the methods chosen justified? - Information provided on strategies ( thick description, triangulation etc.) |  |  |  |  | No- the paper is written as a summary of the group discussion proceedings. There is no detailed description of methods. |
| Were the data analysed appropriately?  Consider the following:   - Is there a clear description of methods used for analysis? - Did more than one researcher analyse the data? If yes, is this reported in detail? |  |  |  |  | The paper only provides a descriptive summary of the barriers and strategies proposed. No details of methods chosen for analysis are available. |
| Are the results generalizable?  Consider the following:   - Do the findings resonate with other similar contexts? - Does the study advance any theoretical understanding or framework? |  |  |  |  | Results are generalizable and themes identified resonate |
| Does the study adequately address potential ethical issues including reflexivity   - Does the research consider the ethical impact of collecting personal experiences and placing them in the public domain? - Does the research consider the fallout on the participant of sharing such experiences (if applicable) |  |  |  |  | Responses are all deidentified- to that extent it protects the participants.  No details on ethics approval or consent available. |
| Overall: is what the researchers did clear? |  |  |  |  | Despite limitations, the paper offers important insights into the barriers for research as well as the barriers researchers from LMICs face when they return to home countries |

1. **Study quality assessment: Franzen et al.**

| **Appraisal questions** | **Yes** | **No** | **Unsure** | **NA** | **Comments** |
| --- | --- | --- | --- | --- | --- |
|  |  |  |  |  |  |
| Was the sample used appropriate for the research question?  Consider if the sample was chosen keeping in mind participant experiences as relevant to the study question, but also based on social affiliations- for e.g. religion, culture, profession etc. |  |  |  |  | Researchers with prior experience or stakeholders with interest in clinical trials identified.  Participants identified using a combination of approaches- search of trial registries and publications, search of the Global Health Trials website and snowball sampling. |
| Were the data collected appropriately?  Consider the following:   - Were the methods for data collection described? - Were the methods chosen justified? - Information provided on strategies ( thick description, triangulation etc.) |  |  |  |  | Yes, a combination of methods were used- interviews, focus group discussions and process mapping.  Methods have been described in detail including a flow or process diagram.  Data collection tools are also described. |
| Were the data analysed appropriately?  Consider the following:   - Is there a clear description of methods used for analysis? - Did more than one researcher analyse the data? If yes, is this reported in detail? |  |  |  |  | Detailed description of analytic methods are available.  Broadly, individual case-analysis followed by cross-analysis.  Transcripts analysed by thematic coding analysis and the conceptual models were based on realist research. |
| Are the results generalizable?  Consider the following:   - Do the findings resonate with other similar contexts? - Does the study advance any theoretical understanding or framework? |  |  |  |  | Yes – pilot done in Ethiopia and then a confirmatory study done in Cameron and finally a comparative analysis performed in Sri Lanka. Wide variety of settings and contexts. |
| Does the study adequately address potential ethical issues including reflexivity   - Does the research consider the ethical impact of collecting personal experiences and placing them in the public domain? - Does the research consider the fallout on the participant of sharing such experiences (if applicable) |  |  |  |  | Broadly, yes.  Ethics approval obtained from all countries involved. Consent- either written or verbal obtained from participants.  Participants’ comfort and preferences respected broadly. |
| Overall: is what the researchers did clear? |  |  |  |  |  |

1. **Study quality assessment: Sawe et al.**

| **Appraisal questions** | **Yes** | **No** | **Unsure** | **NA** | **Comments** |
| --- | --- | --- | --- | --- | --- |
|  |  |  |  |  |  |
| Was the sample used appropriate for the research question?  Consider if the sample was chosen keeping in mind participant experiences as relevant to the study question, but also based on social affiliations- for e.g. religion, culture, profession etc. |  |  |  |  | Yes, efforts made to identify staff working in areas of the hospital involved in trauma care.  A diverse set of participants from 5 hospitals in Tanzania included. |
| Were the data collected appropriately?  Consider the following:   - Were the methods for data collection described? - Were the methods chosen justified? - Information provided on strategies ( thick description, triangulation etc.) |  |  |  |  | Yes, main mode of data collection was the use of focus group discussions.  FGDs were conducted in a conference room away from site of patient care and lasted approximately 60mins. They were audio recorded and continued until saturation was reached. |
| Were the data analysed appropriately?  Consider the following:   - Is there a clear description of methods used for analysis? - Did more than one researcher analyse the data? If yes, is this reported in detail? |  |  |  |  | Yes, well described.  Hybrid thematic data analysis used and included inductive and deductive reasoning.  Codebook developed by one author and then edited along with other authors. This was then pilot tested.  Analysis is appropriately described. |
| Are the results generalizable?  Consider the following:   - Do the findings resonate with other similar contexts? - Does the study advance any theoretical understanding or framework? |  |  |  |  | While the findings do resonate with other low and lower-middle income contexts, a limited number of themes were identified. |
| Does the study adequately address potential ethical issues including reflexivity   - Does the research consider the ethical impact of collecting personal experiences and placing them in the public domain? - Does the research consider the fallout on the participant of sharing such experiences (if applicable) |  |  |  |  | Appropriate ethics committee approvals were sought and obtained. Informed written and verbal consent was also obtained.  However, the authors don’t describe much about how participant confidentiality, comfort, experiences and preferences were respected. |
| Overall: is what the researchers did clear? |  |  |  |  |  |

1. **Study quality assessment: Johnson et al.**

| **Appraisal questions** | **Yes** | **No** | **Unsure** | **NA** | **Comments** |
| --- | --- | --- | --- | --- | --- |
|  |  |  |  |  |  |
| Was the sample used appropriate for the research question?  Consider if the sample was chosen keeping in mind participant experiences as relevant to the study question, but also based on social affiliations- for e.g. religion, culture, profession etc. |  |  |  |  | Participants were a mix of clinicians, researchers and other key stakeholders from the 3 participating sites and the registry coordinating centre |
| Were the data collected appropriately?  Consider the following:   - Were the methods for data collection described? - Were the methods chosen justified? - Information provided on strategies ( thick description, triangulation etc.) |  |  |  |  | Yes, interviews and ethnography used.  Limitation: ethnography component of study not well described. |
| Were the data analysed appropriately?  Consider the following:   - Is there a clear description of methods used for analysis? - Did more than one researcher analyse the data? If yes, is this reported in detail? |  |  |  |  | The methods section describes the framework (Theroretical Domain Framework) and it also states that two researchers independently coded data.  Reasonable amount of information provided in the methods to provide the readers an understanding of what was done. |
| Are the results generalizable?  Consider the following:   - Do the findings resonate with other similar contexts? - Does the study advance any theoretical understanding or framework? |  |  |  |  | Results are generalizable and themes identified resonate |
| Does the study adequately address potential ethical issues including reflexivity   - Does the research consider the ethical impact of collecting personal experiences and placing them in the public domain? - Does the research consider the fallout on the participant of sharing such experiences (if applicable) |  |  |  |  | Responses are all deidentified- to that extent it protects the participants.  Ethics approval taken at all participating sites. |
| Overall: is what the researchers did clear? |  |  |  |  |  |
