# Supplementary material for: Barriers and facilitators to the conduct of critical care research in low and lower-middle income countries: A scoping review
Source: PLoS One. 2022 May 5;17(5):e0266836. doi: 10.1371/journal.pone.0266836 (PMC9071139; doi:10.1371/journal.pone.0266836)
Supplement: S3 Appendix — (PDF) [file pone.0266836.s003.pdf]

Risk of Bias Instrument for Cross-Sectional Surveys of Attitudes and Practices

Contributed by the CLARITY Group at McMaster University

1. Is the source population representative of the population of interest?

Definitely yes (low risk of bias)	Probably yes	Probably no	Definitely no (high risk of bias)

Examples of low risk of bias:

- Selection of target population (either the entire population or a random sample) from a representative population roster such as a national association database

Examples of higher risk of bias ("probably yes" or "probably no"):

- Single centre/city/region study
- Non-random sampling

Examples of high risk of bias:

- Studies where the source population cannot be defined (or enumerated), i.e. any volunteer studies using self-recruitment

2. Is the response rate adequate?

Definitely yes (low risk of bias) | Probably yes | Probably no | Definitely no (high risk of bias)

Examples of low risk of bias:

- High enough response rate to ensure that any differences would be unlikely to affect results (>75%)

Examples of higher risk ("probably yes" or "probably no") of bias:

- > or = 25% missing data but statistical analysis shows no difference in demographic variables that are associated with variability in survey responses between respondents and non-respondents

Examples of high risk of bias:

- Response rate of <50% and no testing done to explore the differences between respondents and non-respondents, or testing indicates that important difference exist

These proportions may not apply to all situations at. At times, lower proportions may be acceptable. At times, higher may be legitimately demanded.

3. Is there little missing data?

Definitely yes (low risk of bias)	Probably yes	Probably no	Definitely no (high risk of bias)

Examples of low risk of bias:

- Less than 10% missing data within questionnaires

Examples of higher risk of bias ("probably yes" or "probably no"):

- Less than 15% missing data within questionnaires

Examples of high risk of bias:

- More than 15% missing data within questionnaires

These proportions may not apply to all situations at. At times, lower proportions may be acceptable. At times, higher may be legitimately demanded.

4. Is the survey clinically sensible?

Definitely yes (low risk of bias) | Probably yes | Probably no | Definitely no (high risk of bias)

Examples of low risk of bias:

- Formal assessment of the comprehensiveness, clarity, and face validity of the questionnaire in a similar population

Examples of higher risk of bias ("probably yes" or "probably no"):

- Formal assessment of comprehensiveness, clarity, and face validity of the questionnaire in a different population

Examples of high risk of bias:

- No evidence that comprehensiveness, clarity, and face validity of the questionnaire have been assessed

5. Is there any evidence for the reliability and validity of the survey instrument?

Definitely yes (low risk of bias) | Probably yes | Probably no | Definitely no (high risk of bias)

Examples of low risk of bias:

- Reliability and construct validity (i.e. convergent and discriminant validity) of the survey have been well-established in a similar population

Examples of higher risk of bias ("probably yes" or "probably no"):

- Some evidence of robust psychometric properties
- Reliability and construct validity of the survey have been well-established in a different population

Examples of high risk of bias:

- No evidence that reliability and construct validity have been established for the instrument
