# Supplementary material for: Barriers and facilitators to the conduct of critical care research in low and lower-middle income countries: A scoping review
Source: PLoS One. 2022 May 5;17(5):e0266836. doi: 10.1371/journal.pone.0266836 (PMC9071139; doi:10.1371/journal.pone.0266836)
Supplement: Attachment — Submitted filename: Response to Reviewer Comments 28 march 2022.docx [file pone.0266836.s006.docx]

**Response to Reviewer Comments**

We thank the Editor and Reviewers for the thoughtful feedback and are grateful for their assistance in improving the manuscript. We respond below- all editorial and reviewer comments are italicized with responses below each comment.

**Additional Editor Comments:**

*Please provide some references for the implications of practice section especially from line 272-278. There are already a number of published studies and systematic reviews on the solutions proposed by the author team.*

Response: This is now done- we have added references 29-34 which support the statements in the ‘implications for practice’ section

**Reviewer 2 Comments:**

*Summary:*

*The authors have responded comprehensively to the comments and incorporated the changes in the manuscript.*

Response: Thank you.

*Minor issues*

1. *In line 134, the context can be modified to read ‘Low and Lower-middle income countries’.*

Response: this is now done.

2. *In line 147 to 148, ‘Study quality was assessed using Cochrane Risk of Bias 2.0 tool…….’*

*I suggest this sentence should not be included since there was no RCTs in the included studies but relevant to be included in the protocol.*

Response: This sentence has now been deleted.

3. *In line 149 to line 150, please check the font size*

Response: thank you- corrected.

4. *In line 196, Please include year of publication in table 1. It helps the reader to interpret the findings appropriately based on the time the research was conducted. It also saves the reader the time and energy taken to go through the list of references to check the year of publication.*

Response: Thank you- agree and added.

5. *Figure 1 is not clear; the words are faint therefore the reader may strain to make sense of it.*

Response: We have now revised this figure for better clarity.
